# Traction-band clip-assisted biliary cannulation for a papilla located in the horizontal limb after total gastrectomy

**DOI:** 10.1055/a-2779-3536

**Published:** 2026-02-03

**Authors:** Haruo Miwa, Yugo Ishino, Kazuki Endo, Ritsuko Oishi, Yuichi Suzuki, Hiromi Tsuchiya, Shin Maeda

**Affiliations:** 1 26437 Gastroenterological Center, Yokohama City University Medical Center, Yokohama, Japan; 2 26438 Department of Gastroenterology, Yokohama City University Graduate School of Medicine, Yokohama, Japan

**Fig. 1:**
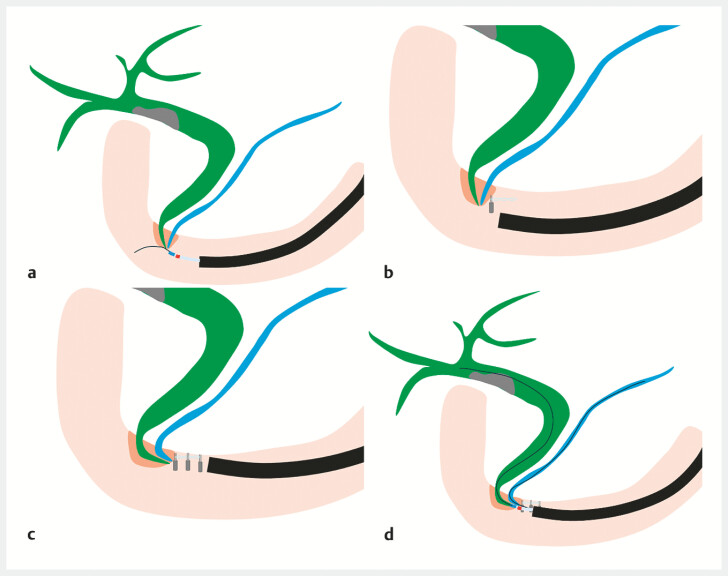
Schema of traction-band clip-assisted biliary cannulation. **a** Biliary cannulation with enteroscopy for a papilla located on the horizontal limb is extremely difficult. **b** A SureClip Traction Band (Micro-Tech) is deployed on the anal side of the papilla. **c** Additional clips are deployed to provide counter-traction. **d** After deployment of the traction-band system, the papilla becomes sufficiently stabilized and biliary cannulation is achieved.

Selective biliary cannulation during balloon enteroscopy-assisted endoscopic retrograde cholangiopancreatography (BE-ERCP) after total gastrectomy can be extremely challenging, particularly when the papilla is located in an anatomically unusual position 1 . Placing a traction-band clip (SureClip Traction Band, Micro-Tech, Nanjing, China) can stabilize the papilla and facilitate successful cannulation 2 3 ; however, this technique has not been reported during BE-ERCP. Herein, we describe a case in which traction-band clip deployment enabled the successful biliary cannulation for a papilla located in the horizontal limb after total gastrectomy ( Fig. 1 , Video 1 ).

Traction-band clip deployment enables successful biliary cannulation for a papilla located in the horizontal limb after total gastrectomy.Video 1

**Fig. 2:**
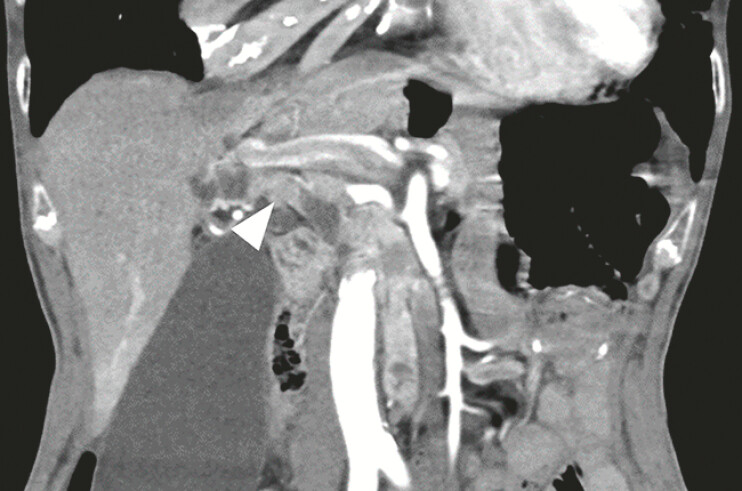
Hypervascular tumor in the common bile duct is revealed on the computed tomographic imaging.

**Fig. 3:**
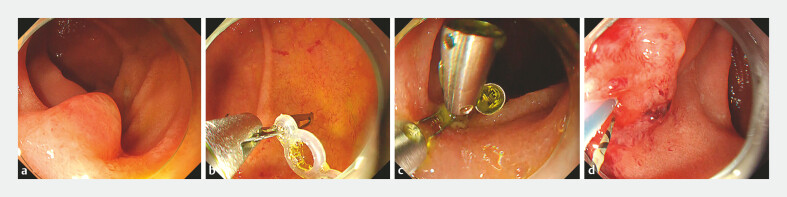
Endoscopic images of the procedure. **a** A papilla is located in the horizontal limb, and biliary cannulation is extremely difficult. **b** A SureClip traction band is attached to the elastic band with two loops. **c** After traction-band clip deployment, stability of the papilla is improved. **d** Selective biliary cannulation is successfully performed under the double guide wire technique.

**Fig. 4:**
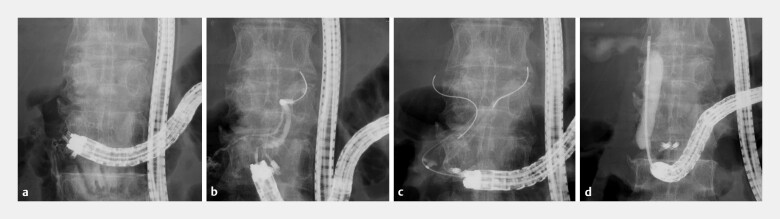
Fluoroscopic images of the procedure. **a** Traction-band clip is deployed at the papilla. **b** A flexible guidewire is advanced into the main pancreatic duct. **c** Biliary cannulation is achieved using the double-guidewire technique. **d** Biopsy of the bile duct tumor with a sheath device reveals adenocarcinoma.

A 78-year-old man who underwent total gastrectomy with Roux-en-Y reconstruction was referred for evaluation of an extrahepatic bile duct tumor. On the computed tomographic image, enhanced tumor was shown in the common bile duct ( Fig. 2 ); therefore, BE-ERCP was planned. When the single-balloon enteroscope (SIF-H290S; Olympus Medical Systems, Tokyo, Japan) was inserted, the papilla was located in the horizontal limb. Despite using a rotatable papillotome, selective biliary cannulation was extremely difficult because of its marked mobility. Therefore, traction-band clip-assisted cannulation was attempted. A SureClip Traction Band was deployed on the anal side of the papilla. The second SureClip was rotated to engage the band, and the enteroscope was gently withdrawn. The clip was then applied to the duodenal wall. Finally, a third clip was deployed to provide stronger counter-traction. After deployment of the traction-band system, the papilla became sufficiently stabilized. A flexible guidewire (Fielder Flex; Asahi Intech, Aichi, Japan) was advanced into the main pancreatic duct. Using the double-guidewire technique, selective biliary cannulation was finally achieved. Biopsy of the bile duct tumor with a sheath device revealed adenocarcinoma ( Fig. 3 and Fig. 4 ).

To the best of our knowledge, this is the first report of selective biliary cannulation using a traction-band clip during BE-ERCP after total gastrectomy. This novel technique may facilitate biliary cannulation in cases where the papilla is located in the horizontal limb.

Endoscopy_UCTN_Code_TTT_1AR_2AB
